# HAEEPGP Peptide Efficiently Prevents Alzheimer's Disease Pathology *in Vivo*

**DOI:** 10.1002/alz70855_097997

**Published:** 2025-12-23

**Authors:** Olga Kechko, Dmitry Yanvarev, Kirill Chaprov, Kristina Mukhina, Alexander Makarov, Vladimir Mitkevich

**Affiliations:** ^1^ Engelhardt Institute of Molecular Biology, Russian Academy of Sciences, Moscow, Russian Federation; ^2^ Institute of Physiologically Active Compounds at Federal Research Center of Problems of Chemical Physics and Medicinal Chemistry, Russian Academy of Sciences, Chernogolovka, Moscow region, Russian Federation

## Abstract

**Background:**

Currently, the only disease‐modifying therapy for Alzheimer's disease (AD) is monoclonal antibodies against beta‐amyloid (Aβ). Such antibodies slow cognitive decline and reduce amyloid burden, however, they do have side effects and cross the blood‐brain barrier (BBB) with low efficacy. Short peptides could be an alternative treatment to high‐molecular weight antibodies. They have been successfully tested against various neuropathologies. In particular, HAEE tetrapeptide is able to cross the BBB, reduces the amyloid plaques, and has no toxic effect *in vivo*. The disadvantage of short peptides is their rapid elimination from an organism. In this work, we added PGP amino acids to the HAEE C‐terminus to stabilize the peptide, studied the pharmacokinetics of HAEEPGP and its ability to suppress AD‐associated pathology.

**Method:**

Direct interaction of HAEEPGP and Aβ peptides was measured by isothermal titration calorimetry. Pharmacokinetics and distribution to tissues of ^125^I labeled HAEEPGP were studied after its intravenous injection in C57BL/6 or 5xFAD mice. HAEEPGP ability to prevent Aβ pathological effect on HMC3 microglial cells was assessed by flow cytometry and IL‐6 ELISA kit. HAEEPGP inhibition effect on AD pathology was studied *in vivo* using two animal models: transgenic *C. elegans* overexpressing human Aβ and 5xFAD mice.

**Result:**

PGP addition to HAEE significantly increased its half‐life time in mice plasma, from 17 to 56±12 min. HAEEPGP propitiously crossed BBB. Its level in the mice brain constantly increased during an hour post‐injection and was higher in 5xFAD than in wild type mice. HAEEPGP bound to Aβ with K_d_ equal to 1.64 μM and even stronger to the isomerized, pathological form of Aβ. Zinc ions significantly enhanced this interaction with both Aβ peptides. HAEEPGP prevented Aβ‐induced inflammation and redox changes in microglial cells, restored lifespan of AD nematodes, and reduced the amount of Aβ plaques and behavioral decline in 5xFAD mice.

**Conclusion:**

We created the stable short peptide HAEEPGP that crossed BBB and specifically bound to Aβ and its isomerized form. HAEEPGP thoroughly inhibited AD‐related abnormalities in cultured microglial cells, AD transgenic nematodes, and 5xFAD mice. Thus, HAEEPGP has promising therapeutic potential for AD. Supported by the Russian Science Foundation (grant #19‐74‐30007).

